# Modelling Variable Fire Severity in Boreal Forests: Effects of Fire Intensity and Stand Structure

**DOI:** 10.1371/journal.pone.0150073

**Published:** 2016-02-26

**Authors:** Yosune Miquelajauregui, Steven G. Cumming, Sylvie Gauthier

**Affiliations:** 1 Centre d’étude de la forêt, Département des sciences du bois et de la forêt, Faculté de foresterie, de géographie et de géomatique, Université Laval, Québec, Québec, Canada; 2 Canadian Forest Service, Laurentian Forestry Centre, Québec, Québec, Canada; Lakehead University, CANADA

## Abstract

It is becoming clear that fires in boreal forests are not uniformly stand-replacing. On the contrary, marked variation in fire severity, measured as tree mortality, has been found both within and among individual fires. It is important to understand the conditions under which this variation can arise. We integrated forest sample plot data, tree allometries and historical forest fire records within a diameter class-structured model of 1.0 ha patches of mono-specific black spruce and jack pine stands in northern Québec, Canada. The model accounts for crown fire initiation and vertical spread into the canopy. It uses empirical relations between fire intensity, scorch height, the percent of crown scorched and tree mortality to simulate fire severity, specifically the percent reduction in patch basal area due to fire-caused mortality. A random forest and a regression tree analysis of a large random sample of simulated fires were used to test for an effect of fireline intensity, stand structure, species composition and pyrogeographic regions on resultant severity. Severity increased with intensity and was lower for jack pine stands. The proportion of simulated fires that burned at high severity (e.g. >75% reduction in patch basal area) was 0.80 for black spruce and 0.11 for jack pine. We identified thresholds in intensity below which there was a marked sensitivity of simulated fire severity to stand structure, and to interactions between intensity and structure. We found no evidence for a residual effect of pyrogeographic region on simulated severity, after the effects of stand structure and species composition were accounted for. The model presented here was able to produce variation in fire severity under a range of fire intensity conditions. This suggests that variation in stand structure is one of the factors causing the observed variation in boreal fire severity.

## Introduction

A fire regime is a quantitative description of the characteristics of the fires that occur in a region [1], including frequency, size, cause, season of burning and the general type of fires (i.e. ground, surface or crown). In boreal North America, the fire regime is characterized by infrequent, high intensity lightning-caused crown fires that are both large and severe [2]. Fireline intensity (fire intensity, hereafter) as defined by [3] is the rate of energy release per unit length of fire front, currently measured in units of kW m^-1^ [4]. It is one of the most important descriptors of fire behaviour to be used in explaining aboveground fire impacts [4,5]. Fire severity, on the other hand, refers to the biophysical or ecological impacts of a fire [6]. Severity is inherently multifactorial. Some aspects that can be readily quantified are the proportion of foliage consumed or killed, and fire induced tree mortality [7,8]. In forested ecosystems, fire intensity has been directly related to scorching height of conifer crowns [9]. Scorch height is defined as the height at which the heat of a fire is lethal to living foliage; it is correlated to the proportion of foliage consumed [10]. Low-intensity surface fires yield lower scorch heights that cause little or no tree mortality, whereas higher scorch heights characteristic of high-intensity fires can kill large trees resulting in nearly 100% tree mortality [11]. In the boreal forest, variation in fire severity can have long lasting effects on the post-fire vegetation community structure and dynamics and on their flammability [2,8].

Boreal tree species have adaptations for survival and persistence in fire-dominated environments. For example, black spruce (*Picea mariana* (Mill.) BSP) and jack pine (*Pinus banksiana* Lamb.), both possess, in different degrees, clumped aerial seedbanks protected by cone serotiny that ensure a seed source for regeneration after a crown fire. However, the two species respond differently (e.g. in terms of mean fecundity and seedling survival rates) to variation in fire severity, as measured by overstory tree canopy mortality and duff consumption [12,13]. These differences in responses to fire severity can in turn affect post-fire regeneration densities and structural development [14]. Low-to moderate-severity fires typically leave most of the large trees alive, which results in structurally complex stands with a broad range of tree diameters [15]. These fires tend to leave on the ground a thick layer of partially charred organic matter, a substrate that negatively affects recruitment and early seedling growth of both species, although with a less important effect on jack pine [8,12]. In contrast, severe fires that kill most trees are likely to regenerate as dense stands with relatively low levels of structural complexity [16]. This is because such fires expose mineral soil, an optimal regeneration seedbed for both black spruce and jack pine [12,13].

Boreal conifer forest stands present a highly flammable configuration of fuels because of their crown architecture (e.g. deep crowns with relatively low crown base heights) [17], their high canopy bulk densities with large amounts of fine twigs and needles, high resin and low foliar moisture contents [2]. From the point of view of the quantity of crown fuels, boreal conifer stands are architecturally easier to burn than other fuel types [18]. During high latitude summers, longer daylight hours and lack of turgid new plant growth are conducive to drying of canopy fuels and thus high fire intensity [19]. It is for these reasons that high intensity crown fires with high flame length, high levels of consumption of the soil organic layers [17], and corresponding high severity have been considered characteristic of these ecosystems [7]. However, recent findings have drawn attention to important variation in fire severity both within and among fires [15,16], even within boreal conifer stands. It is important then to understand the conditions under which such variation in fire severity can arise. One potentially contributing factor is variation in stand structure [9]. In forest sciences, “stand structure” refers to the within-stand distribution of vegetation such as the horizontal and vertical arrangement of trees [20]. Horizontal structure can be measured by stem density and diameter distribution, and vertical structure can be measured by factors such as the height to crown base and tree height [21]. These variables are related to the quantity of available fuels and to their vertical distribution between the surface and top of the canopy [5,22]. Stand structure influences the probability of transition between surface and crown fires [18]. Thus, stand structure is an important factor determining fire behaviour, fire severity, and forest ecosystem resistance and resilience in response to disturbances [22,23,24].

The purpose of this study was to explore and quantify the effects of stand structure and, secondarily, of tree species composition and region, on the stem-mortality component of fire severity within the boreal conifer forests of northern Québec, Canada. We focused on simulated stands of black spruce and jack pine, the two most abundant and important boreal coniferous species [12]. Our objectives were to: 1) quantify the relationship between fire intensity and stand structure on fire severity; and 2) compare severity between black spruce and jack pine stands and among pyrogeographic regions of the study area. We expected that the effect of stand structure on severity will be of greatest importance at lower fire intensities associated with surface fires. High intensity fires almost always become crown fires, where close to hundred per cent tree mortality is the usual outcome. We also hypothesized that severity in pure jack pine stands will be lower than in pure black spruce stands mostly due to dissimilarities in stand structure, including the higher crown base height characteristic of pine. We tested these expectations using a simulation model where fires of varying intensity were applied to a “static” diameter class-structured model of forest stands. Static means there is no growth, mortality or recruitment. Our approach integrates physical models of fire behaviour with empirical models of fire effects (e.g. severity). The model was calibrated with data representative of our study area derived from inventory plots and historical fire intensities (1994–2010) estimated from spatially interpolated meteorological data. This study conducts a simulation experiment that evaluates the effect of fire intensity (reflecting weather) and stand structure variables on fire severity in 1.0 ha patches of mono-specific black spruce and jack pine stands in northern Québec, Canada. The results of these simulations provide insight into the causes of variable fire severity in boreal forests.

## Materials and Methods

### Study Area

**Fig 1:**
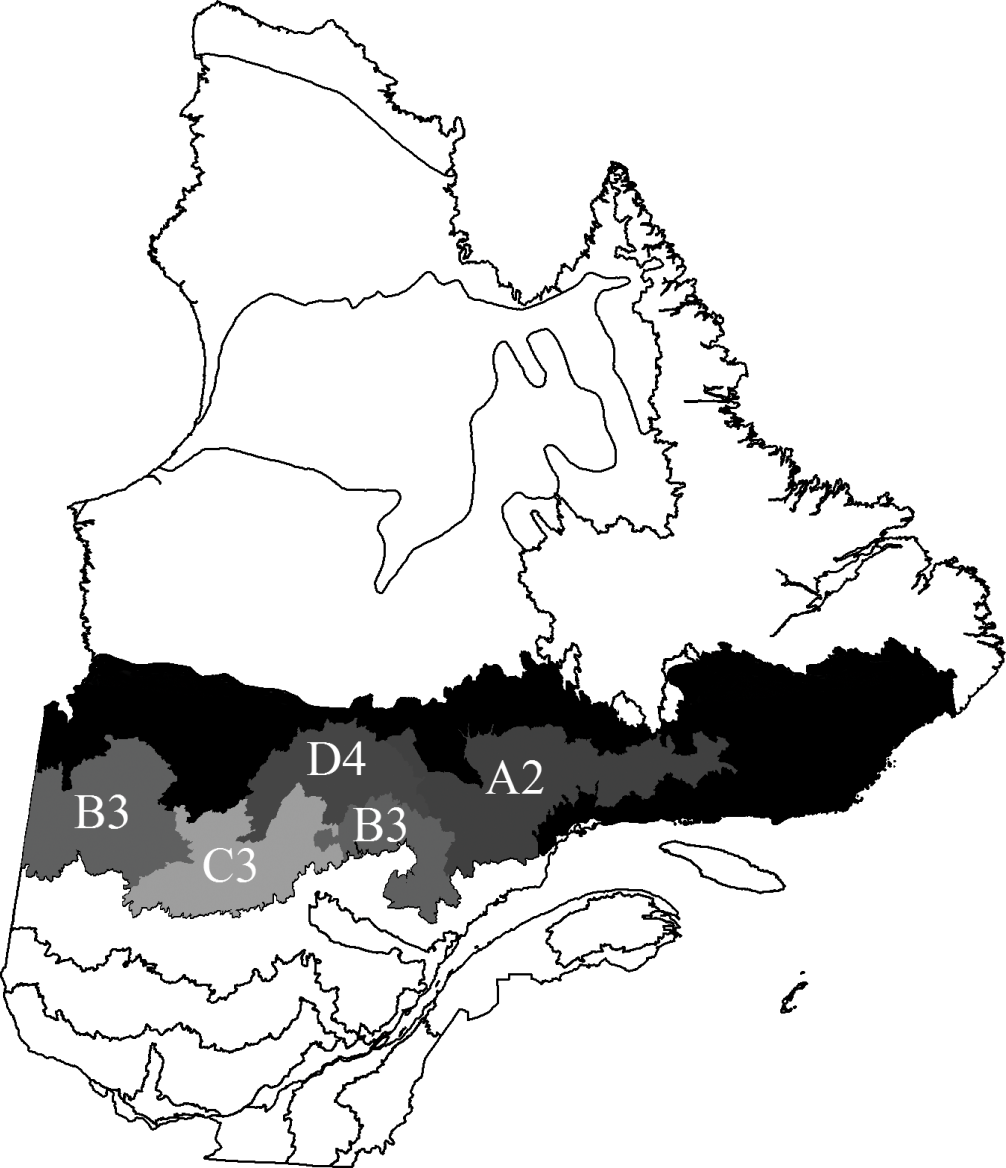
Study area showing the four fire regions chosen. The black spruce feather moss bioclimatic domain in Québec, Canada (shaded area), divided into pyrogeographic regions based on two parameters of the fire regime: the fire cycle and the fire frequency in low, medium and high frequency categories [30]. The four fire regions chosen are shown: A2 (>1100 yrs; low-medium), B3 (500–1100 yrs; medium), C3 (200–500 yrs; medium), D4 (100–200 yrs; medium-high). Map created using ArcGIS 10.0 software.

Our study area is contained within the black spruce feather moss domain (49° to 52°N, 57° to 79°W), of Québec, Canada, a vast region of approximately 412,000 km^2^ [25] (Fig 1). The domain lies within the Canadian Shield, a large area of exposed Precambrian rock [26]. It is characterized by a flat topography with surficial deposits of glacial till that are predominantly thin and discontinuous [27]. Fire is the most common natural disturbance in the domain [17]. Short fire cycles (<180 years) [28] predominate in the western and central portions of the domain, influenced by a continental climate, whereas longer fire cycles (>300 years) are found in the eastern part of the domain due to the humid maritime climate [29]. [30] identified relatively homogeneous pyrogeographic regions -henceforth termed “fire regions”- of Québec forest (Fig 1) based on levels of two fire regime parameters, namely fire cycle, which is estimated as the reciprocal of the mean annual proportional area burned [31], and the number of fires per unit area and time [32]. Four fire regions with contrasting fire regimes within the domain were chosen: A2, B3, C3 and D4 (names represent combinations of the two fire regime parameters following [30]. These regions were expected to capture systematic factors such as climate or soils that potentially influence fire severity independent of stand structure.

### Model Design

**Fig 2:**
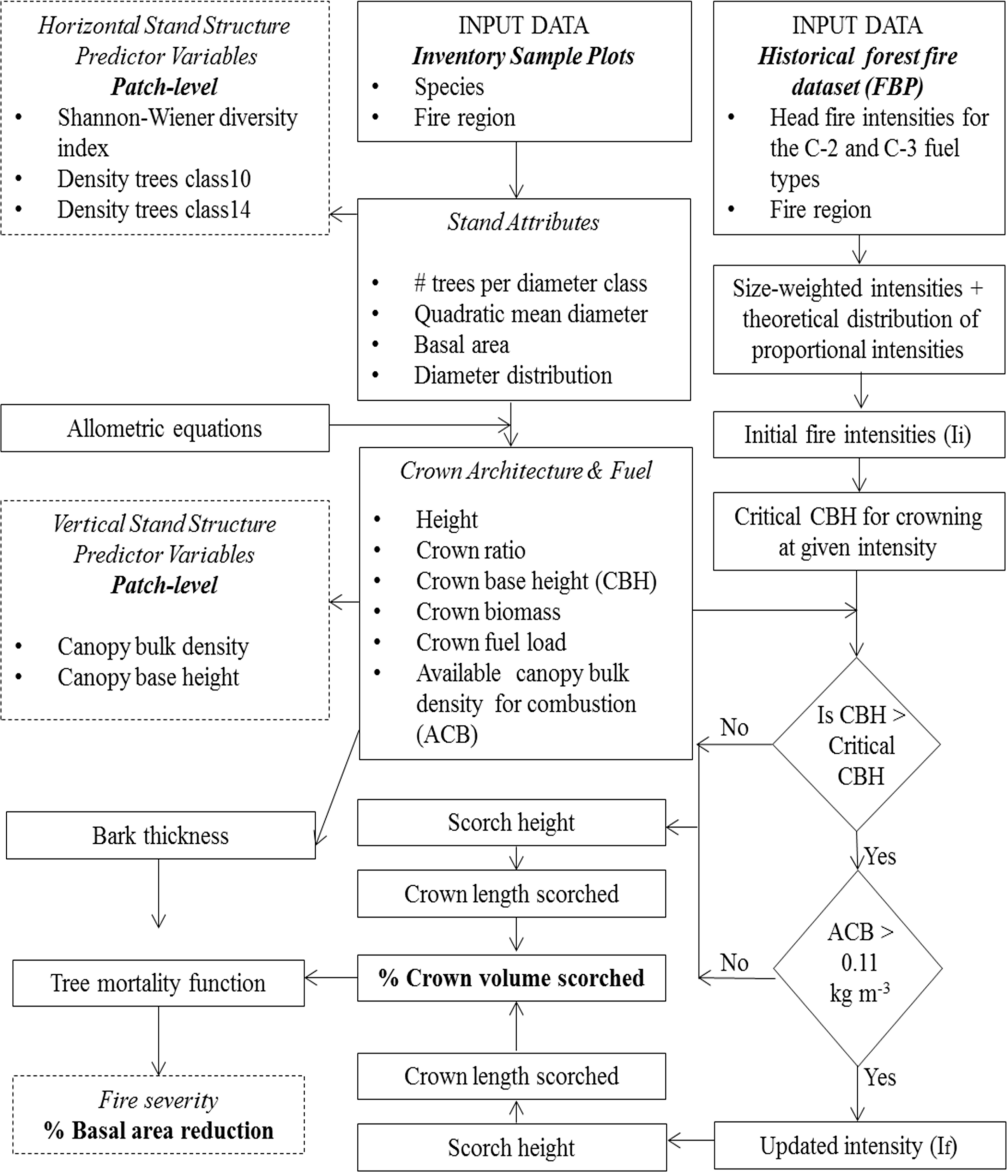
Fire severity model conceptual diagram. Flow diagram of the fire severity model, relating geographically stratified samples of initial fire intensities and forest patch diameter distributions to perform the simulation experiment. Diameter distributions were used to derive fuel and stand structural measures. Crown fire initiation and vertical propagation of a fire was evaluated given the initial fire intensity and the patch canopy fuel characteristics. If crowning occurs, fire intensity is updated and corrected for crown fires [3]. Foliage consumption or scorching is calculated from flame height and foliage profiles. This allows us to calculate size-class specific mortality rates, leading to a patch-level severity measure of basal area loss.

We developed an integrated, size-class structured model of stand structure and fire effects (Fig 2). Our methodology relies on several existing fire modelling systems and empirical allometric equations and models of fire effect and biotic response (Table 1) linking fire intensity, scorch height, the percent of crown scorched and tree mortality to derive one aspect of fire severity, specifically the percent reduction in patch basal area due to fire-caused mortality. The model can simulate the transition from a low intensity surface fire to a high intensity crown fire, as a process of vertical spread into the canopy. The vertical propagation of a fire in the patch is simulated using approximations of the physical conditions limiting the initiation and vertical spread of a crown fire. These critical factors are the surface fire intensity, the canopy base height, and the bulk fuels density in the canopy [18,19,22]. We do not model spatial propagation of a fire line or the interactions between surface fuel bed structure and fire behaviour. The model operates at the 1.0 ha patch level. We defined a “patch” as a homogenous spatial unit with respect to stand composition and structure. A patch is represented by counts of live trees within fifteen diameter at breast height (DBH) classes of 2.0 cm in width, from 1–3 to 29–31 cm. The class quadratic mean diameters were used to calculate stand basal area and other diameter class-level attributes [33] (Table 1). Tree heights and crown ratios were calculated using species-specific allometries [34,35]. Crown base height class was derived from calculated class top heights and class crown ratios [22]. Tree crown biomass (TCB; kg) was calculated using diameter-based crown fuel equations for black spruce [36] and jack pine [37]. Based on [38], crown biomass included the needles and the live branchwood material <0.5 cm in diameter and from 0.5 to 1.0 cm in diameter (Table 1).

**Table 1:** Mathematical equations used in the stand and fire severity model.

Name	Definition	Units	Equation	Reference
**QMD**	Quadratic mean diameter per diameter class	cm	sqrt((X_2_^3^- X_1_^3^)/((X_2_-X_1_)(3))) where X_2_ = upper DBH class limit X_1_ = lower DBH class limit	[33]
**BA**	Basal area of the average tree	m^2^ ha^-1^	(QMD/2)^2^ (3.14)/10,000	-
**H**	Top height per diameter class	m	(**a**) (1.3+1.065) (QMD^0.886) (**b**) (1.3+1.306) (QMD^0.834)	[34]
**CR**	Crown ratio. The ratio of live crown length to tree height	unitless	(**a**)(5.54/(1+((0.007)(BA)))+(4.20)(1-exp((-0.053)(QMD)))-0.45)/10 (**b**) (6.64/(1 + ((0.013)(BA)))+(3.20) (1-exp((-0.052)(QMD))) -0.45)/10	[35]
**TCB**	Tree crown biomass per diameter class. The mass of the living needles and branchwood <0.5 cm and from 0.5 to 1.0 cm in diameter	kg	(**a**) 0.63+ (0.02)(QMD^2.2) (**b**) (0.0079) (QMD^2.41) +(0.0389)(QMD^ 1.729)	(**a**) [36] (**b**) [37]
**FM**	Available fuel masses per diameter class	kg	(TCB)(TEF_*i*_) where TEF_*i*_ = No. trees in each ith DBH class	[38]
**CBH**_**c**_	Critical crown base height	m	(I ^ 0.667) ((460+26)(FMC)) ^1.5 where FMC is the fine moisture content assumed to be 100%	[18,19]
**I**_**f**_	Updated fireline intensity corrected for crown fires	kW m^-1^	(259.833)(H_*i*_ +(H_i_*0.5) ^2.174) where H_i_ is the height of the *i*^th^ DBH class sustaining crowning assuming no effect of wind speed	[3,4,5]
**SH**	Scorch height. The vertical height of the highest point in the crown delineated by yellowing or browning needles	m	(0.1483) (I ^ 0.667) where I is the fireline intensity (I_i_ or I_f_)	[5,11]
**CLS**	Crown length scorched	m	SH-(H-((H)(CR))) if SH>H then CLS = (H)(CR)	[43]
**CS**	Percentage of tree crown volume that is consumed or scorched	%	(100)((CLS/H)(CR))	Modified from [41,43]
**BT**	Bark thickness	cm	(**a**) (0.032)(2.54)(QMD) (**b**) (0.040)(2.54)(QMD)	[43]
**M**	Mortality probability	unitless	(**a,b**) 1/(1+exp(-1.941+6.316 (1-exp(-0.3937)(BT))-(0.000535)(CS^2)))	[41,43,44]
**S**	Live overstory basal area reduction. Measure of fire severity	%	(100) (1- (BA after / BA before))	-
CBH*	Stand mean canopy base height. Average height from the ground to the bottom of the live stand’s canopy	m	sum (((H_*i*_)(1-CR_*i*_))(TEF_*i*_))/sum(TEF_*i*_) where TEF*i* = # trees in each *i*^th^ diameter class	[22]
CFL*	Canopy fuel load	kg ha^-1^	sum((TCB)(TEF_*i*_))	[22]
CL*	Average length of the canopy fuel stratum	m	sum(((H_*i*_)(CR_*i*_))(TEF_*i*_)))/sum(TEF_*i*_)	[22]
CBD*	Canopy bulk density. The available canopy fuel per unit canopy volume	kg m^-3^	CFL/CL	[18,22]
SWDI*	Shannon-Wiener diversity index	unitless	-∑ *p*_*i*_ (ln (*p*_*i*_)) where *p*_*i*_ = relative proportion of trees in each DBH class	[55]

Names, definitions, units, equations, and sources of the variables used in the fire severity model. Equations and parameters are for (a) black spruce and (b) jack pine.

* denotes stand structure variables estimated at patch-level.

The diameter-class specific tree crown biomass values were multiplied by the number of trees in each diameter class to obtain fuel mass load (kg, Table 1). A vertical fuel profile for each patch was obtained by summing fuel masses in thin (1 m) vertical layers along the tree canopy, across all DBH classes and dividing by the volume of that layer (plot area x layer depth) [39]. We computed the “available” canopy bulk density for combustion using the running mean approach [39]. The “available” CBD provides information on the height of the densest layer within the canopy and is an appropriate measure to model crown fire behaviour [40]. When a fire of a given surface intensity (I_i_) is initiated in the patch, there is a minimum crown base height that will allow for vertical propagation of a surface fire into the canopy [18,19,41] (Fig 2, Table 1). If crowning is initiated, the “available” canopy bulk density for combustion is compared to a critical bulk density threshold of 0.11 kg m^-3^ [42], to determine if a crown fire could be sustained. This “available” CBD is a target value for assessing the rate of spread (R_0_) to sustain active crown fires [41] and has been empirically determined by [42] from experimental crown fire data covering a wide range of Canadian boreal coniferous fuel types, and a diversity of crown fuel structures [18,42]; therefore it can be applicable to any fuel type included in the FBP System prone to crown fires. This threshold is supported by similar wildfire case studies [38] and used in other fire behaviour modelling frameworks (e.g. FFE-FVS) [41]. For the purpose of this study, the same CBD threshold was assumed for both the C-2 and C-3 fuel types.

If both conditions apply, crowning is assumed and the fire intensity is updated (I_f_) and corrected for crown fires as suggested by [3,4,5], to reflect a flame length consistent with the top height of the deepest canopy stratum capable of sustaining combustion. This assumes no effect of wind speed on flame’s geometry [4] (Table 1). Otherwise, the initial fire intensity (I_i_) is used for further computations. Scorch height is calculated as a function of intensity, after accounting for vertical propagation [5,11]. The percentage of crown volume scorched per DBH class is determined from calculated scorch height, class top heights and class crown ratios by approximating the crown shape as a cylinder [43,44]. The probability of tree mortality following fire per DBH class was modeled as a function of stem diameter, bark thickness and the percentage of crown volume scorched (Table 1) [44]. The number of trees killed in each DBH class is sampled from a binomial distribution given the predicted class mortality and the number of trees prior to the fire. From this, the model calculates pre- and post-fire basal area. Fire severity is measured as the percent basal area reduction due to mortality (Fig 2; Table 1).

### Historical Forest Fire and Forest Mensuration Data

To run the model we required head fire intensities and tree diameter distributions. Head fire intensities representative of our study area, were selected from an historical forest fire database (1994–2010) provided by the Société de protection des forêts contre le feu (SOPFEU) [45], the province of Québec’s forest fire management agency. Database attributes for each recorded fire include the date when the fire was detected, the location and fuel type at detection, a final size, and the head fire intensity for the first day of burning. These head fire intensities were estimated according to the Canadian Forest Fire Behaviour Prediction (FBP) System from the assigned fuel type, and interpolated local solar-noon temperature, relative humidity, wind speed, and precipitation data [46,47]. The geographical coordinates were used to select fires within one of the four regions (Fig 1). We then classified fires as black spruce or jack pine if their fuel types were C-2 and C-3, respectively. The C-2 fuel type is characterised by pure, moderately well-stocked black spruce stands, with a low crown base. The C-3 fuel type is characterized by pure fully-stocked jack pine stands that have matured to the stage of crown closure closed [46,47]. Fires of other fuel types were excluded, as were fires whose final size was recorded as zero. A total of 1,290 fire records were selected, of which 1,111 were classed as black spruce (C-2), and 179 as jack pine (C-3). SOPFEU is required to actively suppress all fires when first detected [45], and this policy was in place over the entire study interval. Fire management objectives in Québec are that fires be contained within a final size of 3.0 ha [45]. Not all fires can be successfully contained given their size and intensity at initial attack [48]: approximately 43% (n = 556) of the historical forest fires (1994–2010) in our study area exceeded the 3.0 ha target, and can be regarded as having escaped initial attack. We found a significant relationship (*F*_1,1288_ = 6.06, *p* = 0.013) between fire intensity and fire size in our study region, meaning that low intensity fires are associated with smaller fires.

Diameter distributions and stem counts were obtained from Québec’s extensive network of inventory sample plots [49]. The plot data contain descriptive and quantitative information at the plot level (e.g. geographical coordinates, altitude, slope, drainage class, surficial deposit type) and at the tree level (e.g. species and diameter at breast height). Plots are circular with an area of 400 m^2^ and a small circular subplot of 40 m^2^ at the center. Tree species and measured DBH are recorded for all live trees with a DBH greater than 9.1 cm. Height and age are recorded only for a subset of sample trees [49]. Saplings (trees with a DBH lower than 9.1 cm) [49] are measured within the 40 m^2^ subplot. Each inventory plot was associated with a list of measured tree diameters which are binned into the size-class structures. The inventory plot level data was scaled up to 1.0 ha. Inventory plots were spatially stratified by fire regions (Fig 1), and then by soil drainage class and surficial deposit, two soil properties related to surface fuel moisture content that reflect the growing conditions of trees within the stand [50]. Plots with “mesic-glacial till” soils, most characteristic of the domain, were kept for the modelling exercise (6,956 of 11,454). These were then classified by species composition [49]. Mono-specific plots were defined as those where a single species contributed more than 75% of the total basal area. We finally retained 3,428 mono-specific plots of black spruce (n = 3,195) and jack pine (n = 233) from which to sample simulated stands distributed among the four fire regions.

### Simulation Experiment

To evaluate the relationship between stand structure, fire intensity and severity, we simulated the severity of fires of randomly chosen intensity on randomly chosen patches, stratified by fire region and dominant tree species. We ran 3,000 simulations for each combination of four fire regions and two dominant tree species, for a total of 24,000 runs. The replicate plots were sampled randomly with replacement from the subset of inventory plots for the given species within the fire region, and scaled to 1.0 ha. Stand attributes and the horizontal and vertical stand structure variables were calculated for each patch.

Fire intensities were sampled by a three-stage process. First, head fire intensities were selected from the historical forest fire database for the appropriate region and fuel type. Given the association between head fire intensity and fire size in our study area, we used a size-weighted sampling scheme to sample with replacement 3,000 head fire intensity records. For this procedure we considered fires with a minimal final size of 0.1 ha. By accounting for area burned, the low fire intensities that were associated with smaller fires were less likely to be sampled [51]. As a result, we expected the range of intensity values used in the modeling experiment to be more representative of the distribution of intensities over area burned. The estimated head fire intensities represent a mix of surface and crown fires, and overestimate the average fire intensity within the burn, even assuming constant burning conditions and elliptical growth [52]. Fire severity is related to rate of spread [9], which varies along the perimeter of an ellipse, and is asymmetric along the major axis [53]. Under this model of elliptical fire growth, the theoretical distribution of intensity within the burn can be derived from well-established principles of fire behaviour [52]. We used this approach to account for variation in intensity within fires, assuming elliptical shapes [52]. The average length-to-breadth ratios characteristic of jack pine and black spruce wildfires [53] were used to determine the shape of the ellipsoids.

We then sampled 3,000 proportional intensities from the theoretical distribution and multiplied them by the size-weighted head fire intensity to provide a patch-level initial fire intensity (I_i_). For each simulation, an initial intensity was applied to a sampled patch and updated to account for crown fire initiation. Fire severity was then calculated within the sample patch given the fire intensity (Fig 2). Following [54], we classified fires as low, medium or high severity according to severity values of <25%, between 25 and 75%, and >75%, respectively. Although there seems to be no real consensus in terms of the meaning of these categories, such classifications can be useful for forest managers because they can be easily applied in aerial surveys of post-fire crown scorching [54].

### Stand Structure Variables

For each sampled patch, we calculated a set of horizontal and vertical stand structure variables that were used as predictors of fire severity in statistical analysis (Fig 2). We used three horizontal structure variables that have been shown to effectively discriminate among stand structure types within this system [55]. These measures were the Shannon-Wiener diversity index (SWDI) of the diameter-class counts, and the percent density of trees in the 10- and 14-cm diameter classes. The SWDI (Table 1) measures the unevenness of the diameter distribution and can be related to the time elapsed since the last major disturbance [20,21,55,56]. Lower values of this index (1.2–1.7) are characteristic of stands with an even distribution of tree sizes that were affected by a recent disturbance, while higher values (1.8–2.4) [55,56] are characteristic of uneven-sized stands where the time since the stand-initiating disturbance is probably long compared to the lifespan of the dominant tree species. The percentages of live stems in the 10- and 14-cm diameter classes are also important stand structure attributes [55]. An increased density of small stems (e.g. 10–14 cm DBH) within the patch could contribute to an overall increase in fire intensity, thus in severity, as the canopy base height decreases and the abundance of ladder fuels increases [18,42,57].

The vertical stand structure covariates included in the analysis were the patch-level canopy base height (CBH, m; Table 1) and the canopy bulk density (CBD, kg m^-3^; Table 1). These variables are related to the quantity and vertical distribution of fuels within the canopy; therefore are important in estimating the potential of surface fires to transition to crown fires [22,40]. Canopy base height was estimated from calculated class top height and class crown ratios and weighted over diameter classes [22] (Table 1). The canopy bulk density was estimated using the load-over-depth approach by [18,22], that simply divides canopy fuel load by canopy length (Table 1). This straightforward approach allows relatively simple estimation of CBD, without the need to account for individual tree variations [39,58]. To obtain the canopy fuel load (CFL, kg ha^-1^), the tree crown biomass values were multiplied by the number of trees in each diameter class and summed over classes (Table 1). Canopy length, defined as the average length of the canopy fuel stratum, was estimated by subtracting the calculated class crown base from the class top height and weighted over diameter classes [22]. Our patch-level CBD differs from the “available” canopy bulk density used in evaluating crowning, in that the former represents an average across all canopy layers and the latter the variability of canopy fuel over horizontal space [40].

### Statistical Analysis

Associations among the stand structure variables were determined, within species, using the Pearson product moment correlation coefficient (R package “stats”). We tested for differences in the stand structure variables and compared the mean fire severity among the four fire regions and between species using ANOVA’s and Tukey’s HSD tests. We compared historical and initial fire intensities among fire regions and fuel types. To model relationships between fire severity and the covariates of fire intensity, stand structure, species composition and fire region, we used a two-step approach based on nonparametric decision trees. A random forest analysis (RFA; R package “randomforest”) was first used to rank the potential covariates in terms of the strengths of their relationships to the response variable, and select a parsimonious subset based on this ranking. Then, a regression tree analysis (RTA; R package “party”) was used to classify the burned plots into groups of similar severity using the subset of covariates identified in the previous step. Both the RFA and the RTA are non-parametric methods suitable for ecological data with complex non-linear and interacting relationships between the predictors and the response. The random forest analysis outperforms many other statistical methods in terms of classification accuracy and is very useful when, as here, the true model is not known [59].

Once the important variables were identified by the RFA, an implementation of RTA called a conditional inference tree was applied. RTA recursively partitions the data into subsets, called nodes, which are relatively homogeneous in the response. Partitions or “splits” are determined by a threshold value of a single covariate, selected to maximise dissimilarity between the two new nodes. In conditional inference trees, covariates are selected by permutation-based significance tests. This reduces variable selection bias and overfitting [59,60] in comparison to similar methods such as Classification and Regression Trees. Nodes that cannot be further split are called terminal nodes [60]. For each terminal node, we calculated the mean, the median, and the coefficient of variation of fire severity. Separate trees were built for black spruce and jack pine stands. We used R version 2.15.0 [61] for all statistical and graphical analysis.

## Results

**Fig 3:**
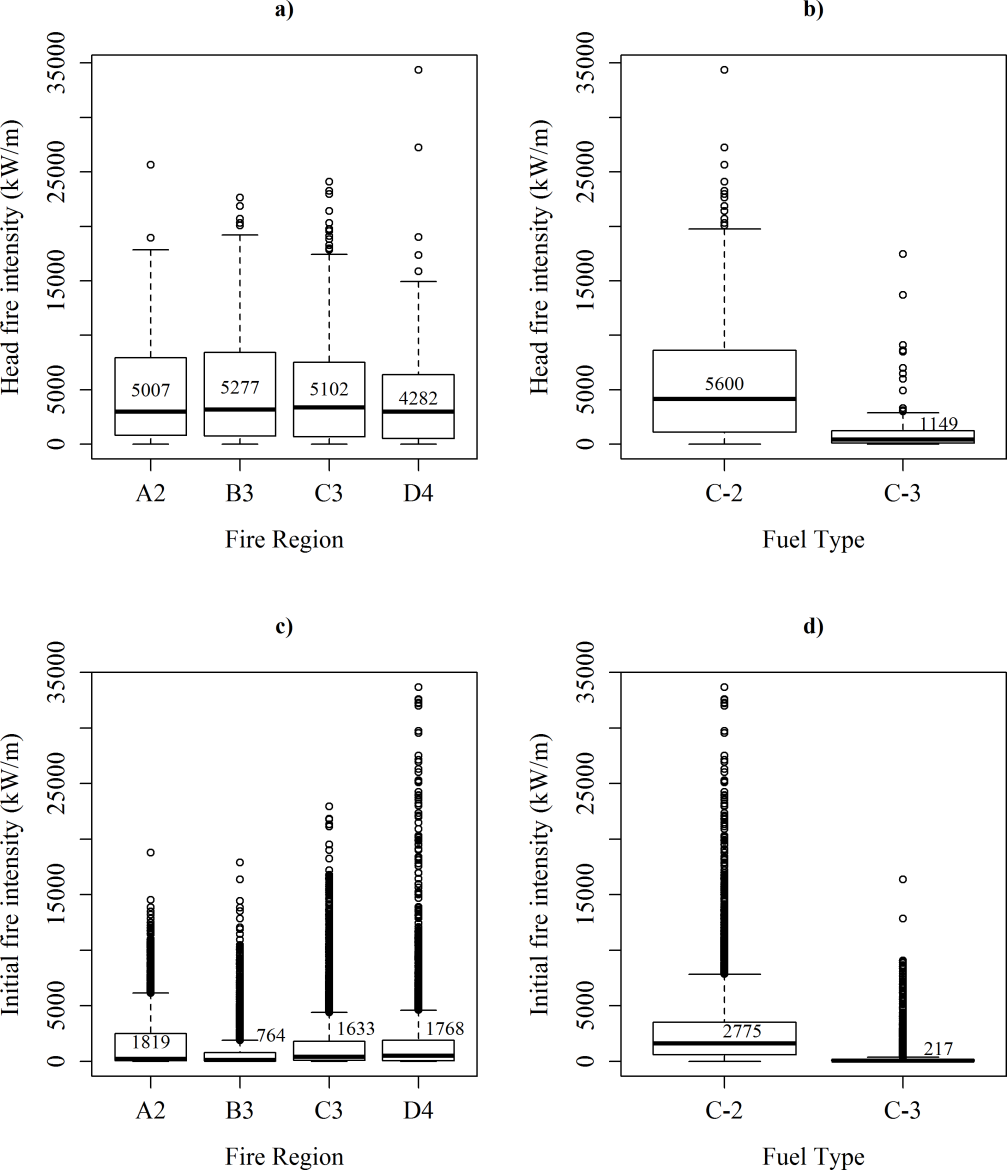
Distribution of the historical and initial fire intensities by fire region and fuel type. Boxplots summarizing the distribution of the recorded historical head fire intensities (kW m^-1^) for a) fire region, b) fuel type, and the distributions of initial fire intensities (I_i_, kW m^-1^) for c) fire region, and d) fuel type. Mean values are shown within the boxes. Boxes represent the inter-quartile ranges; horizontal lines within the boxes represent medians; whiskers extend to the most extreme data point that is no more than 1.5 times greater than the 3^rd^ quartile or less than the 1^st^ quartile. Dots above whiskers represent extreme values.

Historical head fire intensities did not differ among the four fire regions (Fig 3A; *F*_3,1286_ = 2.1, *p* = 0.094) but did differ among fuel types (Fig 3B; *F*_1,1288_ = 122, *p* < 0.001). Mean initial fire intensities (I_i_) differed among fire regions (Fig 3C; *F*_3,23996_ = 194, *p* < 0.001) and fuel type (Fig 3D; *F*_1,23998_ = 6885, *p* < 0.001). For both fuel types, we found a significant and marked negative correlations between the SWDI and the % density of trees in the 10-cm diameter class (r = -0.83 and -0.68, respectively; *p* ≤ .0001), and a weak but significant positive association between the former and the CBH (r = 0.35 and 0.38, respectively; *p* ≤ .0001). Very weak correlations were found for the rest of the horizontal and vertical stand structure variables. A significant species and fire region interaction (*p* ≤ 0.001) was found for the stand attributes listed Table 2.

**Table 2:** Descriptive statistics of the stand characteristics and structure attributes.

Fire region								
Stand and structure attributes	A2		B3		C3		D4	
	*BS*	*JP*	*BS*	*JP*	*BS*	*JP*	*BS*	*JP*
No. sample plots	1007	6	539	25	1184	138	465	64
Total density	4154±2577	1374±417	4250±3408	2400±1335	4209±3304	3244±2853	3987±3030	2132±2160
Basal area	24.6±8.4	14.4±3.2	22.6±10.0	18.1±6.4	22.3±9.6	18.5±7.4	19.4±8.8	9.9±5.1
CBD*	**0.31**±0.14 (a)	0.12±0.03 (e)	0.30±0.18 (a)	0.18±0.10 (d)	0.30±0.17 (a)	0.20±0.12 (c)	0.27±0.16 (b)	0.10±0.07 (f)
CBH*	3.0±0.63 (c)	3.4±0.22 (b)	3.0±0.71 (d)	**3.5**±0.57 (a)	3.0±0.72 (e)	3.4±0.78 (b)	2.8±0.60 (g)	2.8±0.59 (f)
SWDI*	**1.7**±0.32 (a)	1.6±0.31 (b)	1.6±0.40 (c)	1.5±0.31 (e)	1.6±0.42 (f)	1.4±0.46 (f)	1.6±0.39 (d)	1.1±0.45 (g)
% trees class 10*	23.6±15.0 (e)	22.3±16.4 (f)	27.0±19.2 (d)	27.4±15.3 (d)	27.8±19.3 (d)	32.5±23.1 (b)	29.5±18.9 (c)	**35.0**±22.9 (a)
% trees class 14*	17.2±7.1 (a)	16.9±6.3 (ab)	16.0±8.5 (de)	**18.2**±7.4 (a)	15.7±8.7 (e)	16.1±8.9 (cd)	16.8±8.8 (bc)	14.2±12.2 (f)
No. fires ≥ 3.0 ha	50 (35%)	10 (76%)	155 (38%)	16 (44%)	127 (37%)	35 (39%)	134 (59%)	29 (72%)

Descriptive statistics of the stand characteristics and structure attributes (mean±sd) in 24,000 simulated 1.0 ha patches summarized by species and fire region. The number of available inventory sample plots per species and fire region are shown. Different letters (in parenthesis) for the stand structure variables tested (*) represent significant differences within rows obtained from a Tukey’s multiple comparison test (α = 0.05). Highest values for each variable tested are shown in bold. The number of fires in the historical record that escaped the management target size of 3.0 ha for each species and within each fire regions is also reported.

### Variation in Fire Severity among Species and Fire Regions

**Fig 4:**
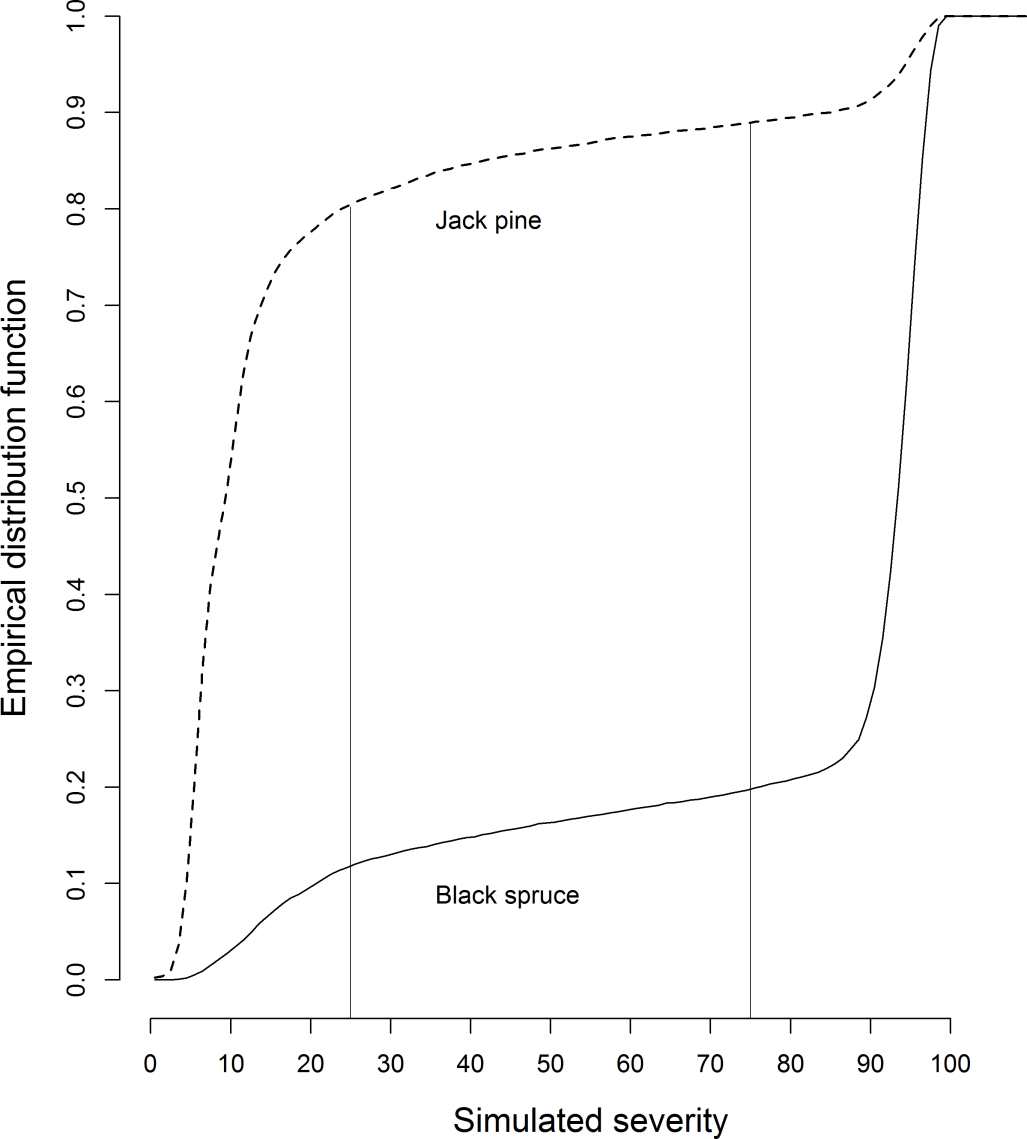
Fire severity empirical distribution function. Empirical distribution function of simulated fire severity, measured as percent reduction in patch basal area, within patches of jack pine and black spruce. Fire severity classes of [54], are delimited by the vertical lines: low severity (<25%), moderate severity (25–75%), and fire severity (>75%).

The proportion of simulated fires that burned at high severity was 0.80 for black spruce and 0.11 for jack pine (Fig 4). This difference was significant (*χ*^*2*^_*1*_ *=* 11577, *p* ≤ .0001). Mean severity was significantly affected by the interaction between species and fire region (*F*_*3*,*23992*_ = 481, *p* ≤ .0001). Mean severity was lower in jack pine stands than in black spruce stands (S1 Fig). Fire region B3 and A2, had the lowest mean severity for black spruce (67%) and jack pine (8%), respectively (S1 Fig).

### Fire Intensity and Stand Structure Effects on Fire Severity

The random forest analysis identified a similar set of important severity predictors for jack pine and black spruce stands. Intensity was by far the most important predictor, followed by the canopy bulk density, the Shannon-Wiener diversity index (i.e. for black spruce), and the percent density of trees in diameter class 10 (i.e. for jack pine). High severity fires were predominantly associated to increased fire intensity, greater canopy bulk density values, higher density of trees in the 10-cm diameter class and lower values of the SWDI. The fire region had the weakest association with severity.

**Fig 5:**
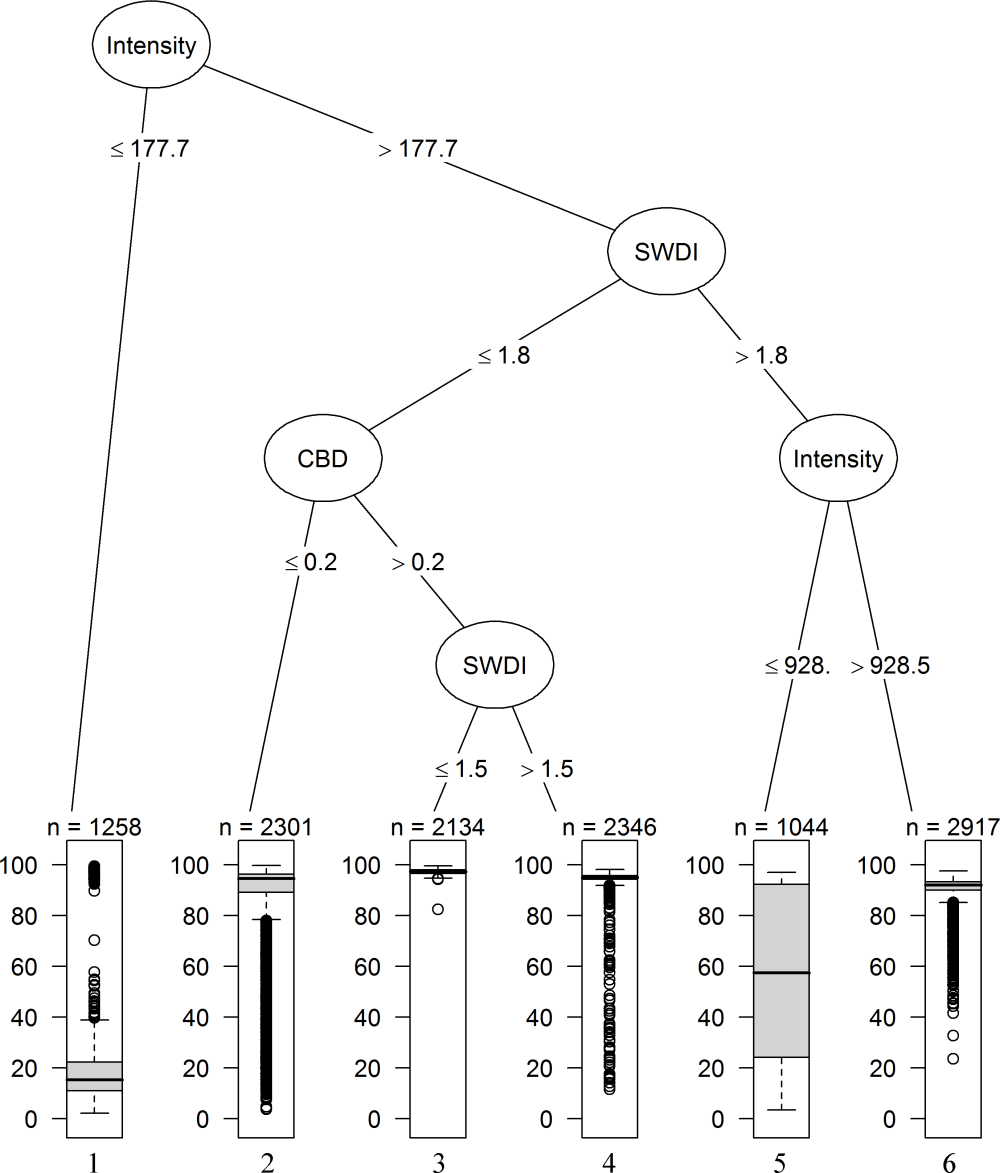
Regression tree for black spruce. Regression tree for simulated fire severity in black spruce patches. The first split in the tree, or the root, is defined by the covariate with the strongest relationship with severity. Box plots at terminal nodes show the distribution of the fire severity data within each branch of the tree. The number of observations within each branch is shown at the top of each boxplot. The total number of simulated fires was 12,000.

The regression tree analysis for black spruce produced a tree with 6 terminal nodes. The model suggests interacting effects of the SWDI, the CBD, and the fire intensity (Fig 5). The first split was determined by intensity ≤ 177 kW m^-1^ (corresponding to a scorch height of 5 m; Table 1). Low fire severity levels (<25%) were associated with intensities below this threshold (terminal node 1). Above this intensity, SWDI showed the greatest association with the response variable. A split at the right side of the tree root was determined by the condition SWDI >1.8 (Fig 5). This corresponds closely to the value distinguishing stands with even (<1.8) from uneven (>1.8) stem diameter distributions. For stands falling on the left side of this node, where SWDI <1.8, the canopy bulk density had the strongest association with the response variable. Stands where this value was less than 0.2 kg m^-3^ (terminal node 2), experienced a median severity of 94% (Table 3). Greater CBD values yielded higher severity fires (>93%, terminal node 3 and 4), and were particularly high (median severity of 97%) when the SWDI was below 1.5 (terminal node 3). This SWDI value lies within the upper bound estimated for stands with even stem size distributions (1.4 ± 0.2) [55].

**Table 3:** Descriptive statistics for each terminal node produced by the regression tree analysis.

	Fire severity descriptive statistics			Fuel characteristics (means)				No. of model runs	Fire intensities (mean±sd)		% simulated patches per fire region			
Terminal node	Mean	Median	C.V	Basal area	CBH	CBD	Total stem density/sapling density	(% total) total = 12,000 per species	Initial intensity (Ii)	Updated intensity (If)	A2	B3	C3	D4
**Black spruce**														
**1**	21.5	15.2	98.7	21.9	2.9	0.30	4270/3234	1258 (10.5%)	83±50	539±3119	10.2	**57.2**	7.70	24.8
**5**	57.6	57.4	57.0	25.5	3.2	0.28	3235/2251	1044 (8.70%)	524±222	8994±14049	14.9	**40.0**	28.9	16.0
**2**	84.8	94.6	25.8	12.8	2.8	0.14	2078/1347	2301 (19.1%)	2921±3162	3303±3855	16.4	22.4	26.2	**34.8**
**6**	90.2	91.9	7.1	25.5	3.1	0.29	3250/2234	2917 (24.3%)	3862±3154	11068±13413	**38.3**	12.4	26.5	22.7
**4**	93.6	95.0	9.1	25.5	3.0	0.34	4754/3435	2346 (19.5%)	3013±2971±	16739±14373	**34.1**	20.2	24.2	21.4
**3**	97.2	97.2	1.0	23.3	2.7	0.42	7330/6148	2134 (17.7%)	2892±3125	18613±15085	19.5	23.9	**30.7**	25.8
**Jack pine**														
**1**	9.8	7.7	92.3	15.7	3.3	0.15	2143/1262	8677 (72.3%)	47±42	127±925	**30.3**	29.5	21.6	18.4
**2**	23.5	13.9	108	14.9	3.2	0.15	2160/1353	1280 (10.6%)	239±51	1566±4260	26.6	8.6	**37.0**	27.6
**3**	67.6	82.4	47.5	10.3	2.3	0.14	4324/3933	397 (3.30%)	88±93	3566±4558	0.0	0.0	**53.6**	46.3
**4**	69.9	84.6	40.3	14.2	3.1	0.15	2507/1724	1645 (13.7%)	1196±1416	3229±6368	1.3	19.9	26.3	**52.3**

Descriptive statistics (fire severity mean, median and coefficient of variation) and mean fuel characteristics (basal area, canopy base height, canopy bulk density, total stem and sapling density) for each of the 6 terminal nodes produced by the regression tree analysis for black spruce and 4 terminal nodes for jack pine. The number of model runs for each node and the percentage of total (%) are shown. The initial fire intensities (Ii) and the updated intensities (If; kW m^-1^) are reported (mean±sd). The proportion of simulated 1.0 ha patches in each fire region within each terminal node are shown. Significant differences in the proportion of patches within terminal nodes and fire regions were found for black spruce (*χ*^*2*^_*15*_ *=* 96.3, *p* ≤ 0.001) and jack pine (*χ*^*2*^_*9*_ *=* 126.0, *p* ≤ 0.001).

For stands falling on the right side of the tree root, that is, where SWDI >1.8 (Fig 5), fire intensity had the greatest association with the response variable. Uneven stands [55] experienced moderate canopy tree fire severities with a median of 57% under intensities > 177 and ≤ 928 kW m^-1^ and corresponding scorch heights between 5 and 14 m, respectively (terminal node 5). Intensities above >928 kW m^-1^ (i.e. scorch height >14 m) in uneven-sized patches, a combination accounting for 24.3% of all simulated black spruce fires, had a median severity of 97% (terminal node 6). Variation in fire severity among the stands within terminal nodes 1, 2 and 5 (CVs. 0.98, 0.25, and 0.57 respectively; Table 3) was greater than among other terminal nodes.

**Fig 6:**
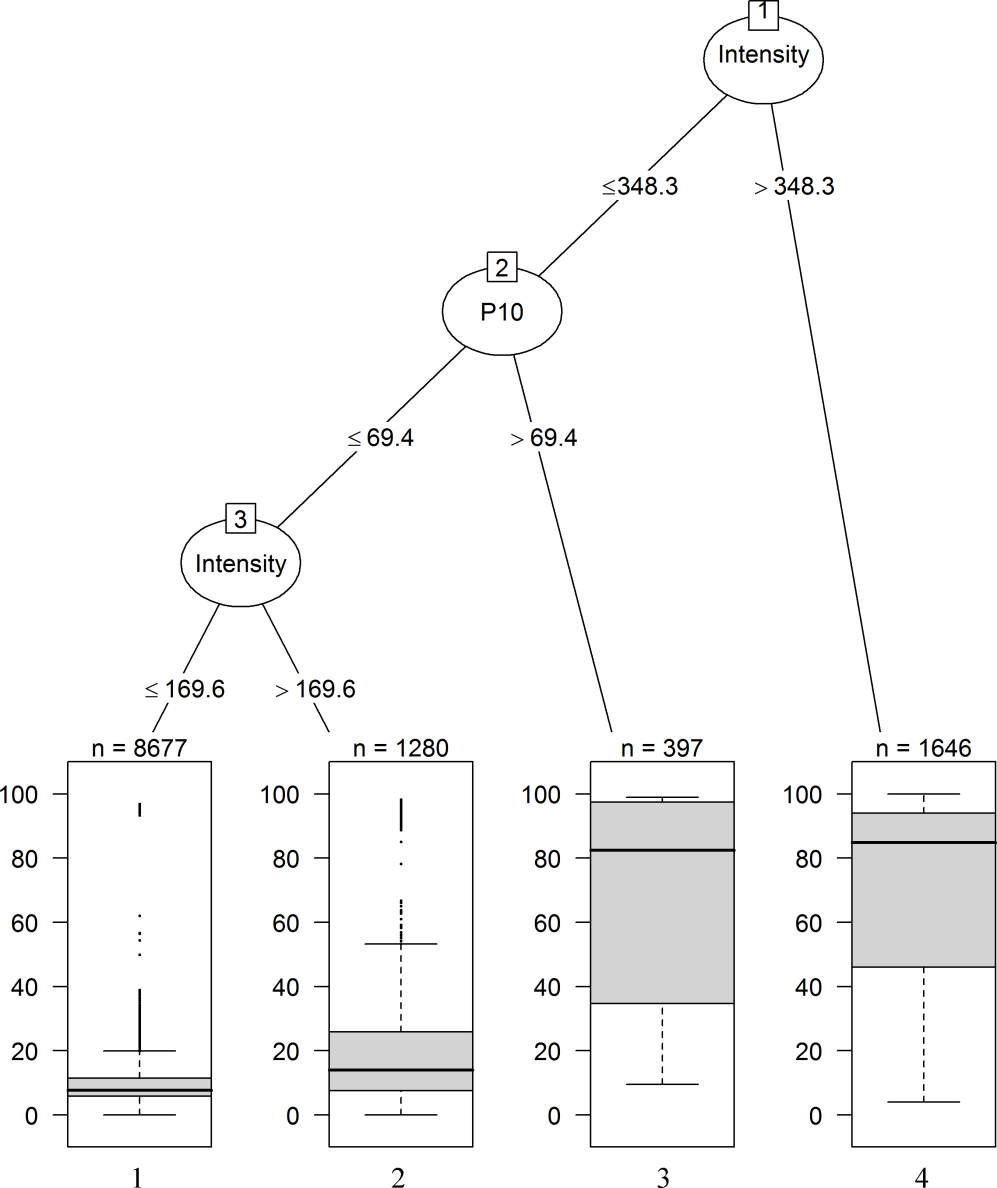
Regression tree for jack pine. Regression tree for simulated fire severity in jack pine patches. The first split in the tree, or the root, is defined by the covariate with the strongest relationship with fire severity. Box plots at terminal nodes show the distribution of the fire severity data within each branch of the tree. The number of observations within each branch is shown at the top of each boxplot. The total number of simulated fires was 12,000.

The conditional regression tree for jack pine is shown in Fig 6, with basic statistics reported in Table 3. Variation in the % density of trees in the 10-cm diameter class produced detectable differences in the resulting severity at intensities below 348 kW m^-1^ (i.e. scorch height of 7 m). Low severity (<25% basal area reduction) was almost always (91% frequency) observed below this threshold. At intensities below 169 kW m^-1^ (i.e. scorch height of 4.5 m), low severity with a median of 7.7% was found in jack pine stands where the % density of trees in the 10-cm diameter class was less than 69% (terminal node 1; Fig 6). High severity fires (>75% basal area reduction) with a median of 82% were observed in stands where this value was greater than 69% (terminal node 3). At intensities above 348 kW m^-1^, high severity (>75% basal area reduction) with a median of 88% was observed (40% frequency; terminal node 4; Fig 6).

## Discussion

We integrated stand structure characteristics derived from inventory plots and fire intensities, within a diameter class-structured fire severity model that accounts for propagation from a surface fire into the canopy. Fire intensities were sampled to as to approximate the unknown distribution of intensities over the historical area burned. The model uses allometric equations to derive stand structure and fuels distribution from diameter-class structures. It uses empirical relations between fire behaviour descriptors and fire effects to evaluate and quantify the influence of the relationship between fire intensity and stand structure on fire severity in 1.0 ha patches of mono-specific black spruce and jack pine stands in northern Québec, Canada.

We found no evidence for a residual effect of pyrogeographic region on simulated severity, after the effects of stand structure and species composition were accounted for. It remains possible that the differences we detected in stand structure among regions reflect differences in fire regime or other geographic factors. The regional implications of these findings would be of interest to pursue in further studies. Our simulations suggest that stand structure is one of the factors causing the observed variation in boreal fire severity. Stand structure exerts its effects primarily through horizontal and vertical structure as reflected in the diameter class distribution and canopy bulk density for black spruce stands and, for pine stands, in the % density of trees in the 10-cm diameter class. We identified thresholds in intensity below which some stand characteristics are conducive to low or moderate severity outcomes. Under high fire intensities associated with crowning and high overstory fuels consumption [19,62], simulated fire severity was independent of stand structure. At lower intensities, we found marked sensitivity to stand structure, and interactions between intensity and structure.

We found that for black spruce, complex structures usually associated to uneven-sized stands [16,55], tended to have lower severity (terminal node 5 in Fig 5). This means that, if an uneven-sized stand was burned at low severity, one might expect that it would be temporarily converted to a relatively even-sized stand of predominantly larger trees. This statement is supported by the fact that approximately 47% (n = 452) of the mono-specific black spruce inventory plots classified as even-sized (n = 951) have basal area values comparable to those found in uneven-sized stands (e.g. mean of 24 m^2^ ha^-1^) [56,63]. As lower severity fires would act as stand-maintaining rather than stand-initiating events, the uneven-sized stands would thus tend to perpetuate themselves as new individuals were recruited beneath the surviving overstory. In other words, stand structure may act as a biotic feedback mechanism, tending to reduce mean fire severity. However, our method for modelling the propagation from surface to crown fire does not account for the spatial distribution of canopy fuel (e.g. clumps of trees) within the patch, which may have an effect on fire behaviour and resulting severity [64]. Therefore, it remains possible that uneven-sized stands with heavy, continuous fuel loading would support the development of severe crown fires under some fire-weather conditions. In that case, the development of heavy fuel loads in such stands would tend to counteract the hypothetical biotic feedback.

The updated intensity values calculated for terminal nodes 1,3,4,5 and 6 of the regression tree for black spruce were substantially higher than the initial intensities (Table 3). However, in the case of terminal node 2, there is a small difference between the initial and updated intensities. Node 2 represents relatively even-sized stands (SWDI<1.8) with low canopy bulk density (CBD<0.2), and they burned primarily at high severity with substantial variation in severity (Fig 5). A possible reason for this small difference between the initial and updates intensities lies in the stand and fuel characteristics (Table 3). For example, node 2 is characterized by mean basal area values of 12 m^2^ ha^-1^, low amounts of available fuel in the canopy and a low density of stems (Table 3), 64% of which is represented by saplings. As the top height of the canopy stratum capable of sustaining combustion decreases, so does the resulting updated intensities (Table 1).

The fire history archives contain estimated head fire intensities at solar-noon on the day the fire started, interpolated from meteorological data [46,47]. These intensities do not apply uniformly to the total area burned by the fire. We used a theoretical distribution of proportional intensity by burned area [52] to correct for this. This correction dramatically reduces the mean intensities relative to the reported values (e.g. in Fig 3). It is possible that in so doing, we underestimate fire severity. To provide the readers with a general insight into the conditions under which variation in fire severity can arise, an additional set of simulations per species was run in which crown fire development was not modelled; that is fire intensity remained fixed regardless of canopy structure. These results are shown in S2 and S3 Figs. Although the regression trees for black spruce and jack pine using the additional set of simulations showed different thresholds in intensity compared to Fig 5 and Fig 6, the conclusion that stand structure explains fire severity within the study region is robust to this correction.

The boreal forest of North America has traditionally been characterised by the presence of large high intensity crown fires which resulted in uniform, near 100% mortality [1,6]. This view has encouraged the adoption of short rotation clear-cut harvesting as the forest management practice most suitable to the region [65]. However, this view of boreal fire regimes has recently been challenged. For example, significant areas of low or moderate severity fire have also been reported in the western boreal forest [16]. Further, the variation in severity implied by our results is consistent with the findings of [66] that revealed heterogeneity in fire impacts, based on visual characteristics of the post-burn landscape within a large wildfire in western Québec. Despite the importance of high intensity crown fires in boreal forest, significant variation in severity clearly occurs. Our results provide a partial biophysical explanation for this phenomenon. Specifically, we show that spatial diversity in stand structure is sufficient to generate variation in fire severity. Other sources of variation in severity include diurnal and longer term variation in fire weather and thus in fire intensity [67]. The relative importance of these and other factors in generating spatial variation in fire severity remains to be elucidated.

### Model Limitations and Extensions

Many models have been developed for simulating patterns of fire effects [68]. Complex models of fire behaviour are capable of describing variation in fire effects in response to stand structure and hourly fire weather conditions, but require very detailed input streams that are not easy to generate [69]. In contrast, the diameter class-structured model presented here uses simple forest fire history and mensuration data, linked with empirical relationships between fire intensity and fire effects. Model uncertainties associated to factors inherent to these relationships are somewhat difficult to quantify because our model integrates so many disparate data sources and past mensuration and modelling studies. We do not think it is necessary to explore these many sources of error in detail for the purposes of the present study. This conclusion might be altered if the precise values of the decision variables in the terminal nodes of Figs 5 and 6 were of great practical importance.

There are some uncertainties in the model assumptions and implementation that could be resolved to improve model predictions. For example, fire severity outcomes are sensitive to assumptions regarding the geometrical representation of the tree’s crown, whether cylindrical, parabolic, or otherwise, because these shapes affect the vertical distribution of fuels [10,58]. Uncertainty in the calculation of crown fuels, namely canopy base height and canopy bulk density could have an effect on our fire severity results because these variables determine the vertical propagation of flaming combustion from the surface to the canopy. Moreover, the calculation of canopy base height is a weighted mean of diameter size-class values. This definition is not unique, and others might lead to different results [40]. Model outcomes could perhaps be improved by integrating multiple data sources and approaches when available, such as individual tree measurements from intensive sampling or Light Detection and Ranging (LiDAR) information [70].

The percentage of crown scorched, an important variable influencing overstory tree mortality and resulting severity, was quantified as function of several variables that were estimated from DBH (e.g. Table 1). The uncertainty involving these quantities and the subsequent effect on the calculation of the percentage of crown scorched, could be reduced by integrating other models that use different crown, stem, and root injury variables to predict tree mortality [71]. We acknowledge that this improvement might increase post-fire mortality prediction accuracy. However, the lack of available models calibrated for black spruce or jack pine made this integration not feasible for the present study. Similarly, post-fire tree mortality is calculated as a function of the scorch height, the percentage of crown scorched, and the bark thickness. Although originally developed for western conifers [44], the generic mortality model such as the one used in our model, are applied in other severity modeling frameworks [41,43]. We found this equation appropriate because they capture the damaging effect of fire on the stem cambium and the loss of foliage in the crown. Moreover, no alternative models have been developed for our region.

Wind speed and direction are critical influences on fire behaviour [1,5,53].Wind speed increases the fire energy output and exposes the unburned fuel to additional radiative and convective heating, thus affecting crown fire initiation and fire spread [72]. However, estimates of wind profiles are complex and tend to vary substantially with height, forest type and stand structure [72]. In addition, a certain proportion of wind momentum is absorbed by the vegetation, resulting in a wind speed reduction [72]. Due to the lack of forest wind speed profile data for our study region, it was decided not to include this interaction in the fire severity model presented here. We also acknowledge that our horizontal and vertical stand structure measures do not provide direct information on the spatial heterogeneity of tree crowns within the patch, which may have an effect on fire behaviour and resulting severity [73]. The recent development of other stand structural diversity indices that combine a mixture of spatial diversity with tree attribute diversity [64], could be included in future versions of the model, if sufficient data was available. However, investigating the effect of spatial heterogeneity of forest fuels within the patch was outside the scope of this study.

We think the most significant sources of uncertainty lies in the sampling of fire intensities used to derive the biological responses. Our raw data were head fire intensities from recent historical fires, estimated from interpolated meteorological data. These intensities represent a mix of surface and crown fires. The distribution of fire intensities used to drive the ecological model was derived from these raw data in several steps. Firstly, we used area-weighted sampling to increase the representation of intensities associated with larger fires [51]. We then applied a distribution of [52] which relates head fire intensity to the distribution of intensities over the areas burned by a single fire. Finally, the intensities could be increased if a crown fire were to develop. The intention of all these steps was to better approximate the unknown distribution of intensities over the historical area burned. We note that the main result of this study, namely that stand structure mediates fire severity within the study region was robust to the way we generated the sample of fire intensities.

In this paper, we evaluated mono-specific patches, but there is no fundamental reason why the model could not be adapted to incorporate two or more tree species. It would also be feasible to incorporate other ecological processes. For example, simulation of snag provision and downed dead wood could be incorporated using empirical falldown and decay rates [74,75]. In fact, we will show in a subsequent paper how a simple matrix model of carbon pool dynamics [76] can be easily coupled to the present model to simulate ecosystem carbon fluxes under alternate fire regimes.

### Implications for Forest Management

Spatial and temporal variation in severity within fires can have long-lasting impacts on the stand structure and species composition of post-fire communities, and on the frequency and effects of future disturbances [6]. Fire severity can affect the amount, nature, and successional trajectory of regenerating vegetation [77], alter post-fire tree fall patterns and decomposition rates of snags [78], affect nutrient cycling [6] and modify the carbon stocks and fluxes of fire-prone ecosystems such as the boreal forest of Canada [7]. All of the factors are of importance in forest management, especially where forest landscape management intends to emulate natural disturbance regime by maintaining a mosaic of stand structures within the forest landscape [79]. Understanding the heterogeneity in fire severity patterns can also have direct, operational implications. For example, fire severity classifications can be used to evaluate prescribed fire success, to assess rehabilitation potential and mitigation of burn impacts [68]. Linking severity information to post-fire vegetation conditions could also improve estimates of future timber volume [80,81].

In this study we addressed near-mono specific stands of black spruce and jack pine, stands associated with the C-2 and C-3 fuel types, respectively. These are two of the sixteen fuel types considered by the FBP System, both of which are well represented in Québec and throughout the boreal Canada [47,82]. Although the FBP System fuel type classification is not intended to cover all variation in black spruce and jack pine stands, our study suggests that stand structure limits fire severity within the C-2 and C-3 fuel types under conditions of low and high-intensity surface fires [19,62]. In Québec, the classification of forest stands as fuels according to the FBP System include variables such as species composition, ratio of softwoods, height and age classes and drainage type [83]. The fire intensity thresholds found in this study could help refining fuel types by integrating measures of stand structure.

## Supporting Information

S1 Fig Fire region and species interaction effect. Interaction plot showing the effect of fire region and species on the mean fire severity with 95% confidence intervals. Different letters represent significant differences between the means obtained from a Tukey’s multiple comparison test (α = 0.05).
(TIF)

S2 Fig Regression tree for black spruce. Regression tree for simulated fire severity in black spruce patches, without simulating crown fire development. The first split in the tree, or the root, is defined by the covariate with the strongest relationship with fire severity. Box plots at terminal nodes show the distribution of the fire severity data within each branch of the tree. The number of observations within each branch is shown at the top of each boxplot. The total number of simulated fires was 12,000.
(TIF)

S3 Fig Regression tree for jack pine. Regression tree for simulated fire severity in jack pine patches, without simulating crown fire development. The first split in the tree, or the root, is defined by the covariate with the strongest relationship with fire severity. Box plots at terminal nodes show the distribution of the fire severity data within each branch of the tree. The number of observations within each branch is shown at the top of each boxplot. The total number of simulated fires was 12,000.
(TIF)
