# Supplementary figures and images for: Modelling Variable Fire Severity in Boreal Forests: Effects of Fire Intensity and Stand Structure
Source: PLoS One. 2016 Feb 26;11(2):e0150073. doi: 10.1371/journal.pone.0150073 (PMC4769146; doi:10.1371/journal.pone.0150073)

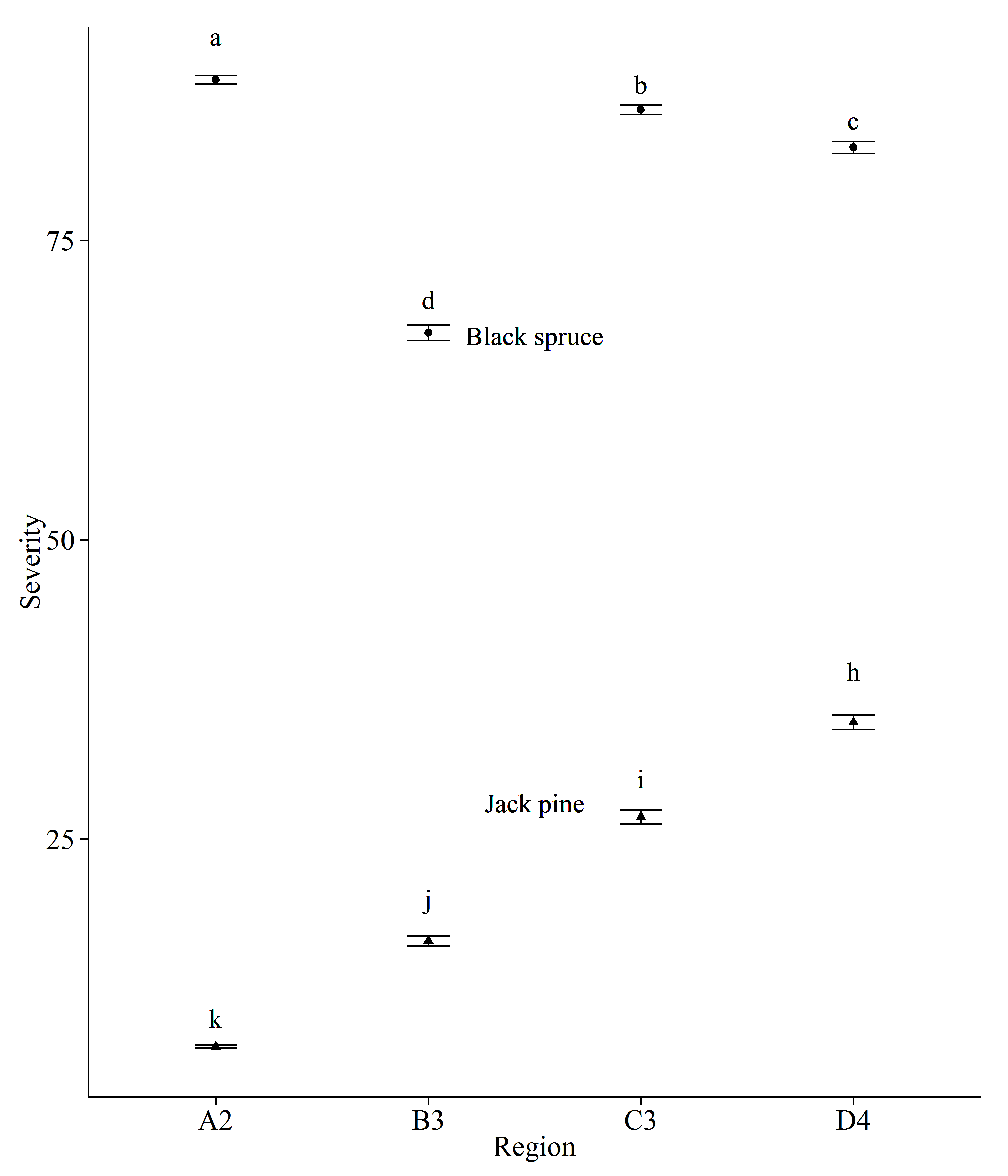

Supplement: S1 Fig — Interaction plot showing the effect of fire region and species on the mean fire severity with 95% confidence intervals. Different letters represent significant differences between the means obtained from a Tukey’s multiple comparison test (α = 0.05). (TIF) [file pone.0150073.s001.tif]

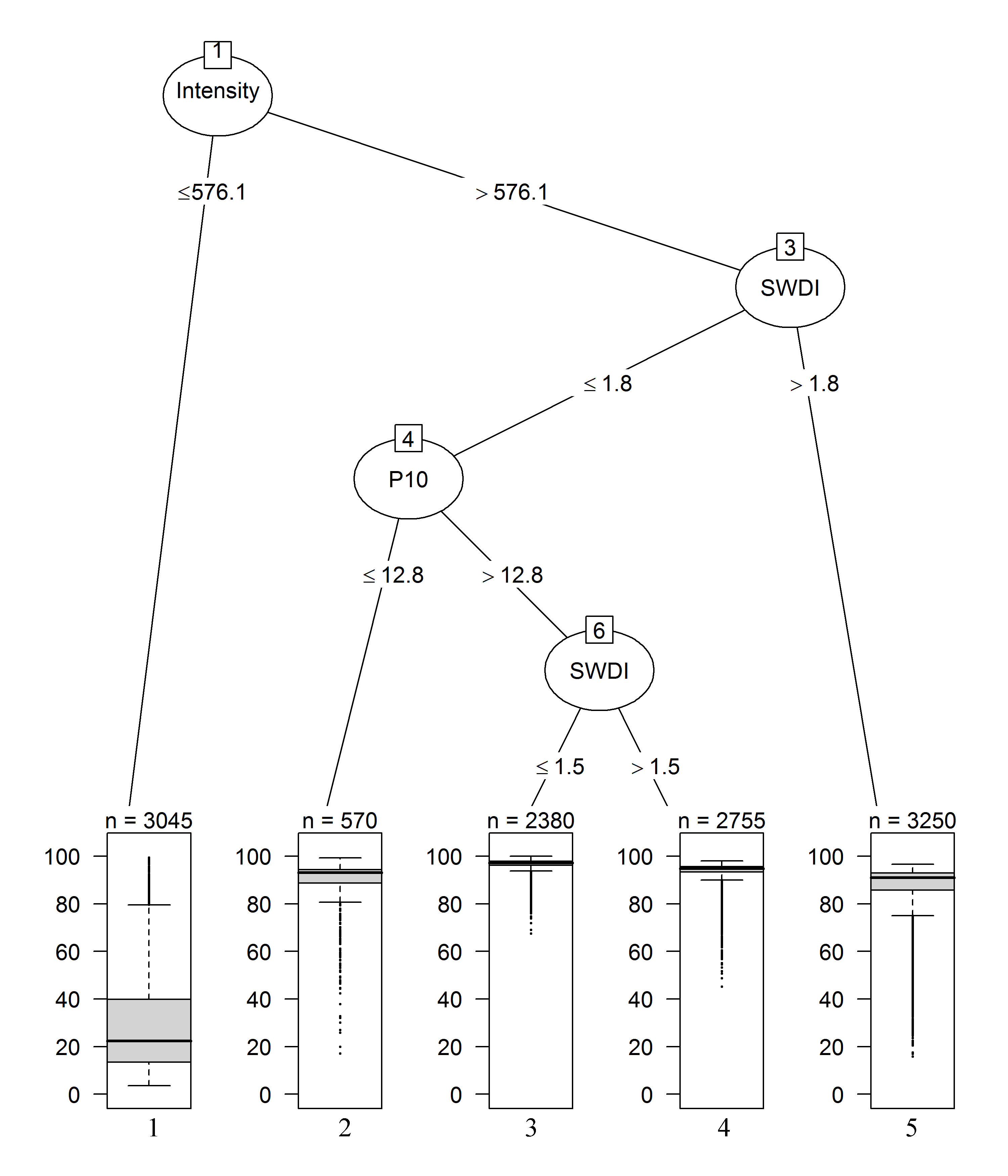

Supplement: S2 Fig — Regression tree for simulated fire severity in black spruce patches, without simulating crown fire development. The first split in the tree, or the root, is defined by the covariate with the strongest relationship with fire severity. Box plots at terminal nodes show the distribution of the fire severity data within each branch of the tree. The number of observations within each branch is shown at the top of each boxplot. The total number of simulated fires was 12,000. (TIF) [file pone.0150073.s002.tif]

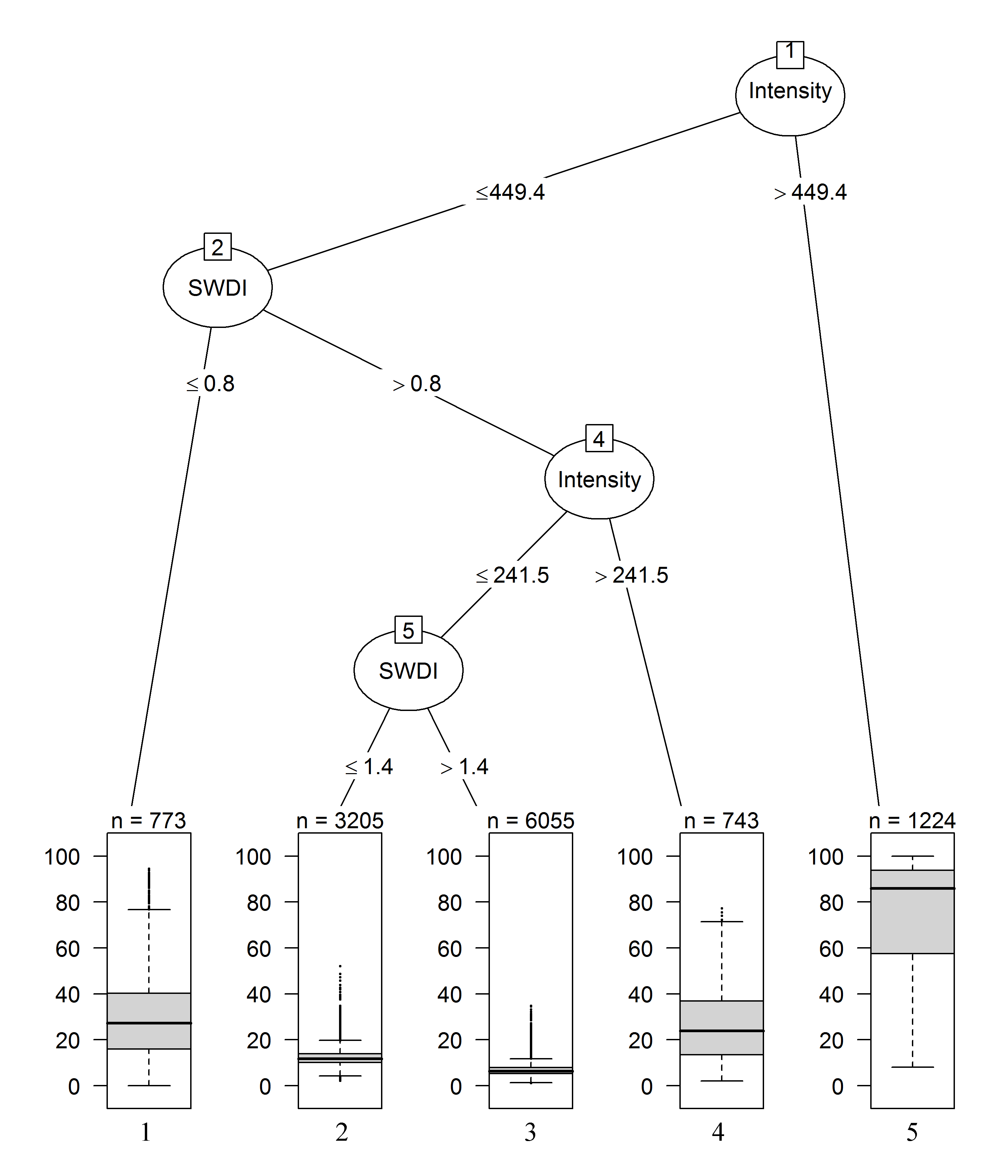

Supplement: S3 Fig — Regression tree for simulated fire severity in jack pine patches, without simulating crown fire development. The first split in the tree, or the root, is defined by the covariate with the strongest relationship with fire severity. Box plots at terminal nodes show the distribution of the fire severity data within each branch of the tree. The number of observations within each branch is shown at the top of each boxplot. The total number of simulated fires was 12,000. (TIF) [file pone.0150073.s003.tif]
